# DEVELOPMENT AND EVALUATION OF A WEB-BASED DECISION AID FOR LUNG CANCER SCREENING FOR OLDER CHINESE AMERICANS

**DOI:** 10.1093/geroni/igac059.2243

**Published:** 2022-12-20

**Authors:** Chien-Ching Li, Alicia K Matthews, Xiaojun Gao, Krystal Cheung

**Affiliations:** Rush University, Chicago, Illinois, United States; University of Illnois at Chicago, Chicago, Illinois, United States; University of Illinois at Chicago, Chicago, Illinois, United States; University of Illinois at Chicago, Chicago, Illinois, United States

## Abstract

Low dose computed tomography (LDCT) lung cancer screening is effective in reducing lung cancer mortality. In the United States, Chinese Americans are disproportionately impacted by lung cancer compared to other Asian subgroups. Our prior study showed that over 25% of older Chinese male smokers met eligibility for LDCT screening based on the CMS guidelines. However, in general, cancer screening rates among Chinese Americans are lower compared to other Americans. Therefore, we developed a culturally and logistically web-based decision aid (DA) for LDCT screening “Lung Decision Coaching Tool (LDC-T)” to improve the older Chinese American's knowledge of lung cancer and screening and to facilitate the decision making with the physician. Compared to traditional paper-based DA, the LDC-T DA has key elements including video, pack-year calculator, eligibility determination, and visual aids to support comprehension and shared decision-making. We conducted acceptability and usability tests among 22 older Chinese American smokers. The tool was highly received by participants for its usefulness and ease to use. Over 80% reported the way of information presented in LDC-T is "good or very good" and the length of the information is "just right". Overall, the majority were satisfied with the LDC-T DA (95%) and agreed that the tool includes sufficient information for people to make the screening decision (90%) and prepare shared decision making with their healthcare providers (81%). Our study results will inform strategies for reducing lung cancer disparities among Chinese Americans and other underserved smokers thus filling important gaps in the literature.

